# Differences in haemosporidian parasite prevalence and diversity in migratory and resident birds of prey species revealed by a non‐invasive sampling method

**DOI:** 10.1002/ece3.11038

**Published:** 2024-03-13

**Authors:** Dovilė Bukauskaitė, Deivis Dementavičius, Saulius Rumbutis, Rimgaudas Treinys

**Affiliations:** ^1^ Nature Research Centre Vilnius Lithuania; ^2^ Tadas Ivanauskas Zoological Museum Kaunas Lithuania

**Keywords:** *Haemoproteus*, *Leucocytozoon*, parasites, *Plasmodium*, raptors

## Abstract

Avian haemosporidian parasites are widespread globally and infect numerous wild bird species. However, they have primarily been studied in passerine birds. Accordingly, the prevalence and diversity of these parasites in birds of prey remain understudied. This lack of research is primarily due to the international protection status of many of these species, their sparse distribution across ecosystems and difficulty to capture in the wild. The aim of this study was to examine the prevalence and diversity of haemosporidian parasites in two species of birds of prey, namely white‐tailed eagle and lesser spotted eagle. To achieve this, a non‐invasive approach was employed, involving the extraction of DNA from blood spots present in moulted feathers. Freshly moulted feathers were collected from the ground under nests or within the nests of these birds during the breeding season. A visible blood spot located at the junction of the calamus and rachis was removed and fixed in SET buffer for molecular analysis. The identification of haemosporidian parasites (*Plasmodium*, *Haemoproteus* and *Leucocytozoon*) was conducted using PCR‐based methods. Overall, parasite DNA was successfully detected in shed feathers that were kept in their original form at least decade prior to analysis. Among the studied individuals, resident white‐tailed eagles showed significantly lower infection rates of haemosporidian parasites compared to migratory lesser spotted eagles. A total of nine genetic lineages of haemosporidian parasites were detected, with seven of them being new to science based on partial sequences of the *cytb* gene. Additionally, the phylogenetic relationships among these new lineages and previously described ones were established. These findings highlight the suitability of non‐invasive sampling for investigating the prevalence and diversity of haemosporidian parasites in wild birds of prey populations. Moreover, this approach holds promise for studying other challenging‐to‐reach and protected bird species. According to our research, there is a greater chance of finding haemosporidian parasites in freshly gathered feathers.

## Graphical abstract

This study describes a new method to investigate haemosporidian parasites in difficult to access birds of prey. Using this method, we determined that resident white‐tailed eagle individuals were significantly less infected with haemosporidian parasites than migratory lesser spotted eagle. This non‐invasive method provides an opportunity to investigate haemosporidian parasites of difficult to access bird groups, like birds of prey or other protected bird species.

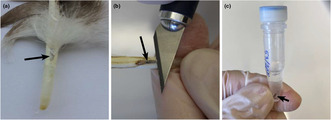

## 1 INTRODUCTION

Avian haemosporidian parasites are widely distributed and infect various bird species (Valkiūnas, 2005). Since these parasites can cause severe pathologies or even mortality in birds (Donovan et al., 2008; Garvin et al., 2003), it is crucial to understand the patterns of infection. Research on these parasites mainly focuses on aspects such as diversity, prevalence, phylogenetics and host specificity among wild birds (Clark et al., 2014; Dimitrov et al., 2010; Ishtiaq et al., 2007; Latta & Ricklefs, 2010; Neto et al., 2015; Szymanski & Lovette, 2005; Yoshimura et al., 2014). However, most of these studies have been dedicated to passerine birds. Research on the diversity and prevalence of parasites in wild populations of birds of prey (Accipitriformes) has received less attention. Investigating parasites across different taxonomic groups of birds is necessary to better understand the broader context of host–parasite interactions (Harl et al., 2022; Lotta‐Arévalo et al., 2023).

### 1.1 Difficulties in studying parasites in wild birds of prey

Birds of prey are long‐lived and are apex predators, resulting in their low densities across many ecosystems. Furthermore, these birds are constrained by habitat alteration and destruction, intentional killing, electrocution, poisoning and climate change (McClure et al., 2018). They have a high sensitivity to environmental contamination, which has led to population crashes in past decades (Helander et al., 2008). Due to historical and ongoing anthropogenic pressures, many species of birds of prey avoid humans and are currently classified as rare, decreasing, threatened or near extinct. As a result, many of these species are internationally protected (McClure et al., 2018). All of the aforementioned factors contribute to the difficulty in sampling blood from free‐living adult birds of prey in their natural habitats. Non‐invasive sampling methods for studying parasites hold promise but are rarely applied, if used at all. This difficulty in blood sampling has led to a limited number of studies investigating parasites in wild‐caught birds of prey (Chakarov & Blanco, 2021; Morel et al., 2021; Sehgal et al., 2006; Svobodová et al., 2023; Tinajero et al., 2019). While some research on parasites in birds of prey species has been conducted in zoos and rehabilitation centres (Chagas et al., 2017; Ciloglu et al., 2016; Huang et al., 2020; Krone et al., 2008; Outlaw & Ricklefs, 2009), it is important to note that in zoos, birds are more easily exposed to local blood‐sucking insects, which can then interact with free‐living birds. This exposure may then influence the natural spread of parasites (Chagas et al., 2017). However, investigating parasites in birds of prey in zoo and rehabilitation centre settings may not accurately reflect the actual patterns of parasite transmission occurring in wild populations.

### 1.2 The use of feathers in birds of prey research

Naturally shed feathers of birds of prey, collected non‐invasively, find wide application in various research areas, including bird species identification (Rudnick et al., 2007), genetic analysis of hybridization between ecologically similar species (Väli et al., 2010), examination of individual physical traits such as body size (Lõhmus & Väli, 2004), individual identification, assessment of spatial behaviour, estimation of population size (Bulut et al., 2016; Rudnick et al., 2005, 2008) and the detection of harmful substance contamination on individuals (Baker et al., 2017; Jaspers et al., 2011). Due to territorial behaviour, extended lifespans of individuals and nest site fidelity observed in many birds of prey species, it is possible to collect these shed feathers under the nests during breeding season when reproductive output is being monitored. The shed feathers, particularly the larger ones such as primaries, secondaries and tail feathers, contain traces of dried blood within the shaft, serving as a source from which DNA can be extracted (Väli et al., 2010). This method may also provide insights into the presence of blood parasites in these birds. In the present study, we used non‐invasively collected feathers as a source of blood from two species of birds of prey: the white‐tailed eagle *Haliaeetus albicilla* and the lesser spotted eagle *Clanga pomarina*. Both eagle species belong to the order Accipitriformes and sympatrically breed in the temperate forests of central‐eastern Europe. Our aim was to assess and estimate the prevalence and diversity of haemosporidian parasites in wild populations of these eagles. Both white‐tailed and lesser spotted eagles are territorial species. Pairs of mated individuals may occupy the same nest built within the canopies of trees for several consecutive years. Feathers that are shed naturally by adult birds during the period of nestling rearing are readily available on the ground under or within nests. Both eagle species share certain characteristics, including deferred reproductive maturity, which is typically reached after at least 4–5 years, and a maximum life span exceeding two to three decades (Böhner & Langgemach, 2004; Helander & Stjernberg, 2003). Both species also overlap in their habitat requirements (Treinys et al., 2011) and therefore occupy nests even in the same forests (Dementavičius et al., 2019). The white‐tailed eagle is a resident species primarily inhabiting temperate and northern regions of Europe (Helander & Stjernberg, 2003), while the lesser spotted eagle migrates annually to winter grounds in southeastern Africa, returning to Europe each spring (Meyburg, 2021).

Using a non‐invasive sampling method, we tested the following hypotheses: (1) shed feathers of birds of prey can serve as a suitable source of blood for molecular haemosporidian parasites research. This suitability is attributed to their proven effectiveness in extracting DNA from bird hosts (Bello et al., 2001), which consequently implies that the DNA of parasites should be also extractable; (2) the success of DNA extraction from both the hosts and the parasites will decrease as the time interval between the collection of shed feathers and blood analyses increases. In other words, given that DNA tends to degrade through time if dried blood is incorrectly buffered (Freed & Cann, 2006), we expect that the feathers analysed within the same year of collection will be a superior source of DNA material compared to feathers collected 5 or 10 years ago and stored without buffers; and (3) the prevalence and diversity of parasites will be higher in the long‐distance migrant lesser spotted eagle compared to resident white‐tailed eagle. Additionally, we investigated the phylogenetic relationships between newly detected lineages and other known *Plasmodium* spp., *Haemoproteus* spp. and *Leucocytozoon* spp. lineages from the GenBank and MalAvi (Bensch et al., 2009) databases.

## 2 MATERIALS AND METHODS

### 2.1 Feather sampling

Between 2010 and 2020, data on nesting site locations and occupancy events of white‐tailed and lesser spotted eagles were collected in Lithuania (55°18′ N, 24°01′ E), located in central‐eastern Europe, to the east of the Baltic Sea. Observations of eagle hunting activities, territorial behaviour and food deliveries were made using binoculars and a spotting scope. These observations took place from spring (March) through summer (August) from vantage points offering clear, unobstructed views of tree‐covered areas. Based on these observations, forest patches were targeted and subsequently checked for nest occupations during the summer or the autumn–winter seasons, when nest visibility was enhanced due to the absence of tree leaves. Both bird of prey species often reuse the same nests year after year (Bergmanis et al., 2019; Dementavičius & Treinys, 2009), which allowed for previously known nests to also be checked for summer occupancy. White‐tailed eagle nests with nestlings were visited in May–June, while successful nests of lesser spotted eagles were primarily inspected in July. Methods for the in‐situ estimation of occupied territories of breeding pairs and the assessment of breeding output for each species are described elsewhere (Treinys et al., 2011). Climbing up to the nests was usually necessary to ring the nestlings. During the nest checking process, freshly moulted feathers (i.e. feathers moulted during the same season as were collected) of adult birds were collected from the nests, on the ground under the nests or from nearby perching trees. We separated these freshly moulted feathers from the feathers left from the previous season based on the conditions of the shafts; because of long exposure to rain and snow, old feathers shafts are typically matte‐whitish or matte‐greyish and partly or heavily decomposed what opposite to freshly moulted feathers shafts in semi‐translucent and elastic condition. The collected feathers, mostly contour (wing and tail) feathers, were assigned to either white‐tailed eagles or lesser spotted eagles based on species‐specific feathers size, colouration and patterning. Collected feathers were labelled with the date of collection as well as the corresponding pair and/or territory identity (Table S1).

After fieldworks, collected feathers were then left to dry at room temperature. Thereafter, feathers were stored in their original shape and condition in a dry place at room temperature without any treatment until analyses. The final sample for this study comprised 90 feathers, representing 45 individuals of white‐tailed eagles and 45 individuals of lesser spotted eagles. For each eagle species, we selected the 15 feathers found within or under the nests occupied in the 2010, 2015 and 2020 field seasons. We used only one feather sampled from the unique breeding territory for the entire period for representing only one individual in our sample. First, we avoided to resampling feathers of the same individuals in different years given that both eagle species have long lifespans and strong breeding territory fidelity; therefore, it is possible for the same member of a pair to reproduce in the same breeding territory for more than a decade (Helander & Stjernberg, 2003; Meyburg et al., 2004). Second, we were limited to distinguish whether the few feathers found during the same breeding territory checking belong to the same individual (i.e. could be shed by female, male, both adults of the pair or even by stranger conspecifics as reported by Meyburg et al., 2007). Additionally, we analysed 12 feathers obtained from taxidermized lesser spotted eagles collected in Lithuania between 1914 and 1932 and which are currently stored in Tadas Ivanauskas Zoological Museum in Kaunas, Lithuania.

### 2.2 Sample preparation for DNA analysis

**FIGURE 1:**
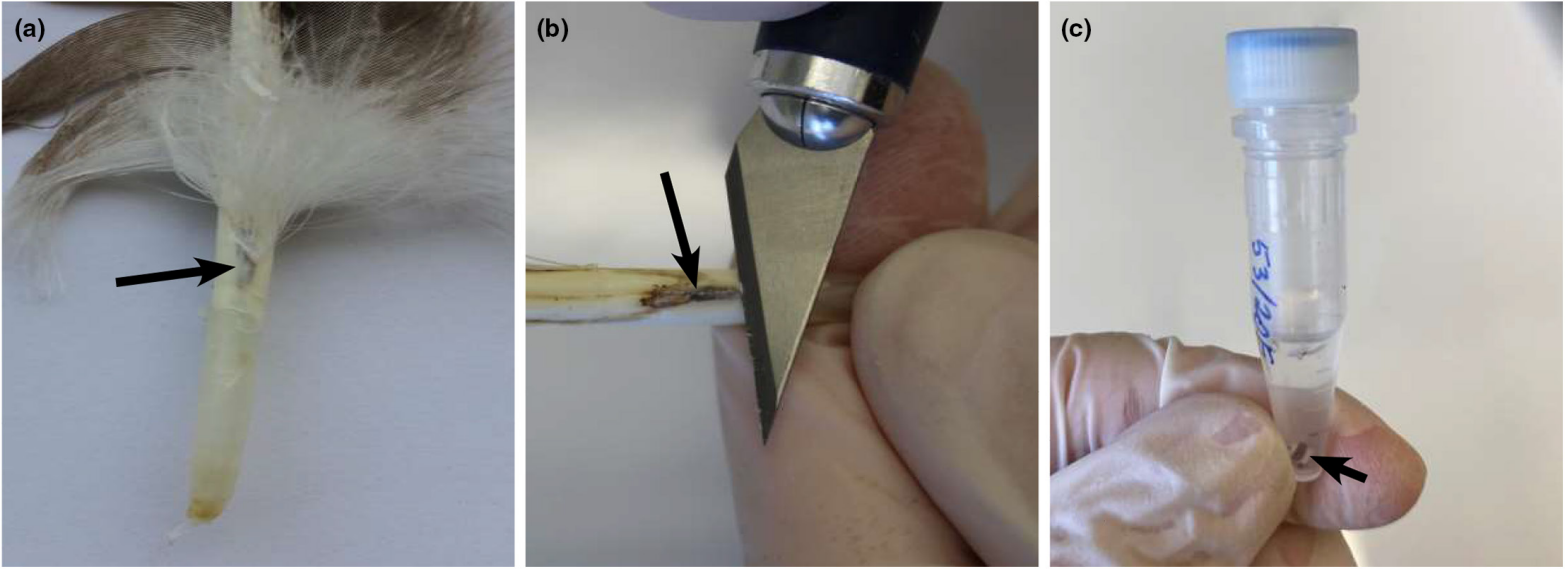
Extraction of dried blood from feathers; (a) a feather with a blood spot; (b) cutting of the dried blood from the feather with a scalpel; and (c) fixing the dried blood in SET buffer. Long arrows indicate dried blood, and short arrows indicate dried blood with inner parts of the superior umbilicus and particles of the rachis and calamus.

Feathers containing visible blood spots at the juncture of the calamus and rachis (i.e. superior umbilicus) were selected for DNA extraction (Figure 1). The region surrounding the blood spot was cleared of down feathers. Then, the area was wiped with 70% ETOH and delicately sliced using a scalpel. The sliced portion was placed into an Eppendorf tube containing SET buffer (0.05 M Tris, 0.15 M NaCl, 0.5 M EDTA, pH = 8.0). Accordingly, dried blood together with the inner part of the superior umbilicus and particles of the rachis and calamus were fixed. It was not possible to exclusively extract the dried blood due to its adherence to the structural components of the feather; any attempt to isolate the dried blood could potentially dissipate and contaminate the samples. Consequently, samples fixed in SET buffer were stored in a freezer at −20°C. All instruments used during sample preparation were disinfected and sanitized via fire and 70% ETOH. After processing each feather, workbench surfaces were disinfected with 70% ETOH.

### 2.3 Molecular methods

#### 2.3.1 Polymerase chain reaction and sequencing

The DNA from the dried blood samples fixed in SET buffer was extracted using standardized ammonium acetate method (Sambrook & Russell, 2001), and no mechanical disruption of fixed blood was used. The concentration of extracted DNA was measured using the Nanophotometer P330 (Implen GmbH, Germany). Samples with DNA concentrations exceeding 50 ng/μL were diluted (≤50 ng/μL DNA) using 1× TE buffer. A nested polymerase chain reaction (PCR) protocol was used to amplify a fragment of the parasites' mitochondrial cytochrome *b* gene (*cytb*). The initial PCR used the primer pair Plas1F/HaemNR3, while the second PCR used the primer pair 3760F/HaemJR4. These primers are designed to amplify haemosporidian parasites in birds of prey. They amplify a 542 base‐pair (bp) fragment of the *cytb* gene found in all three parasite genera: *Plasmodium*, *Haemoproteus* and *Leucocytozoon* (Pérez‐Rodríguez et al., 2013). The amplification conditions used were the same with those described elsewhere (Waldenström et al., 2004). The total volume of the PCR mix was 25 μL, including 12.5 μL of DreamTaq Master Mix (Thermo Fisher Scientific, Vilnius, Lithuania), 8.5 μL nuclease free water, 1 μL of each primer and 2 μL of template DNA. To identify potentially false amplifications, both negative (nuclease free water) and positive (a sample infected with *Plasmodium* sp. parasites) controls were included. The amplified products were evaluated by running 2 μL of the final PCR product on a 2% agarose gel. Amplified samples were sequenced from 3′ and 5′ ends using the Big Dye Terminator V3.1 Cycle Sequencing Kit and ABI PRISM™ 3100 capillary sequencing robot (Applied Biosystems, Foster City, California). Resulting sequences were aligned and examined using Geneious Prime® 2023.1.2. The obtained sequences of the *cytb* gene fragment were trimmed to the standardized length of 478 bp to facilitate comparison. Instances of double base calling in the electropherograms were interpreted as co‐infections.

#### 2.3.2 Phylogenetic analysis

To determine the phylogenetic relationships among new parasite lineages, we constructed two Bayesian phylogenetic trees using both newly obtained sequences and sequences of haemosporidian parasites (described to species level) sourced from GenBank (National Library of Medicine, Bethesda, Maryland, https://www.ncbi.nlm.nih.gov/genbank/). Sixteen *Leucocytozoon* spp. lineages and 4 new (newly obtained) *Leucocytozoon* spp. lineages were used. The tree was rooted with *Haemoproteus minutus* (hTURDUS2). For the second tree, we used 16 *Plasmodium* spp., 51 *Haemoproteus* spp. and 5 lineages obtained from this study. The tree was rooted with *Leucocytozoon* sp. (lSISKIN1). The best fit model (GTR + I + G) was suggested by the jModeltest‐2.1.10 software (Darriba et al., 2012; Guindon & Gascuel, 2003). The phylogenetic tree was run in Geneious Prime® 2023.1.2. with the MrBayes plugin v3.2.6 (Ronquist & Heulsenbeck, 2003). The analysis was run for 6 million generations, with a sampling frequency of every 100th generation. As part of the ‘burn‐in’ process, 25% of the initial trees were discarded before the construction of the consensus tree. The MEGA 11.0.13 software (Tamura et al., 2021) was used to calculate genetic distances.

### 2.4 Statistical analysis

A generalized linear model (GLM) with a ‘binomial’ error structure and a ‘logit’ link function was used to assess whether the probability of infection by blood parasites differed between the eagle species and whether the age of the sampled feathers influenced the quality and viability of the source DNA. Within the GLM, the response variable was ‘infection by blood parasites’, which was a binary variable with the values 0 (indicating no detection of parasite DNA) and 1 (indicating the presence of parasite DNA). Two categorical variables were included in the GLM as explanatory variables: ‘eagle species’ with values ‘Clanga’ and ‘Haliaeetus’ and ‘age of feather’ with values ‘2010’, ‘2015’ and ‘2020’. To determine whether the GLM with both categorical explanatory variables provided a better fit to the data compared to a GLM with only an intercept, we compared the Akaike information criterion corrected for small sample sizes (AICc) of these two GLMs. All regression analyses were performed in the R statistical environment (version 3.6.0; R Core Team, 2019), with the following packages used: *lme4* (Bates et al., 2015), *MuMIn* (Bartoń, 2019), *sjmisc* (Lüdecke, 2018), *sjPlot* (Lüdecke, 2019a), *sjlabelled* (Lüdecke, 2019b) and *ggplot2* (Wickham, 2016).

## 3 RESULTS

### 3.1 Prevalence and diversity of haemosporidian parasites in birds of prey

**FIGURE 2:**
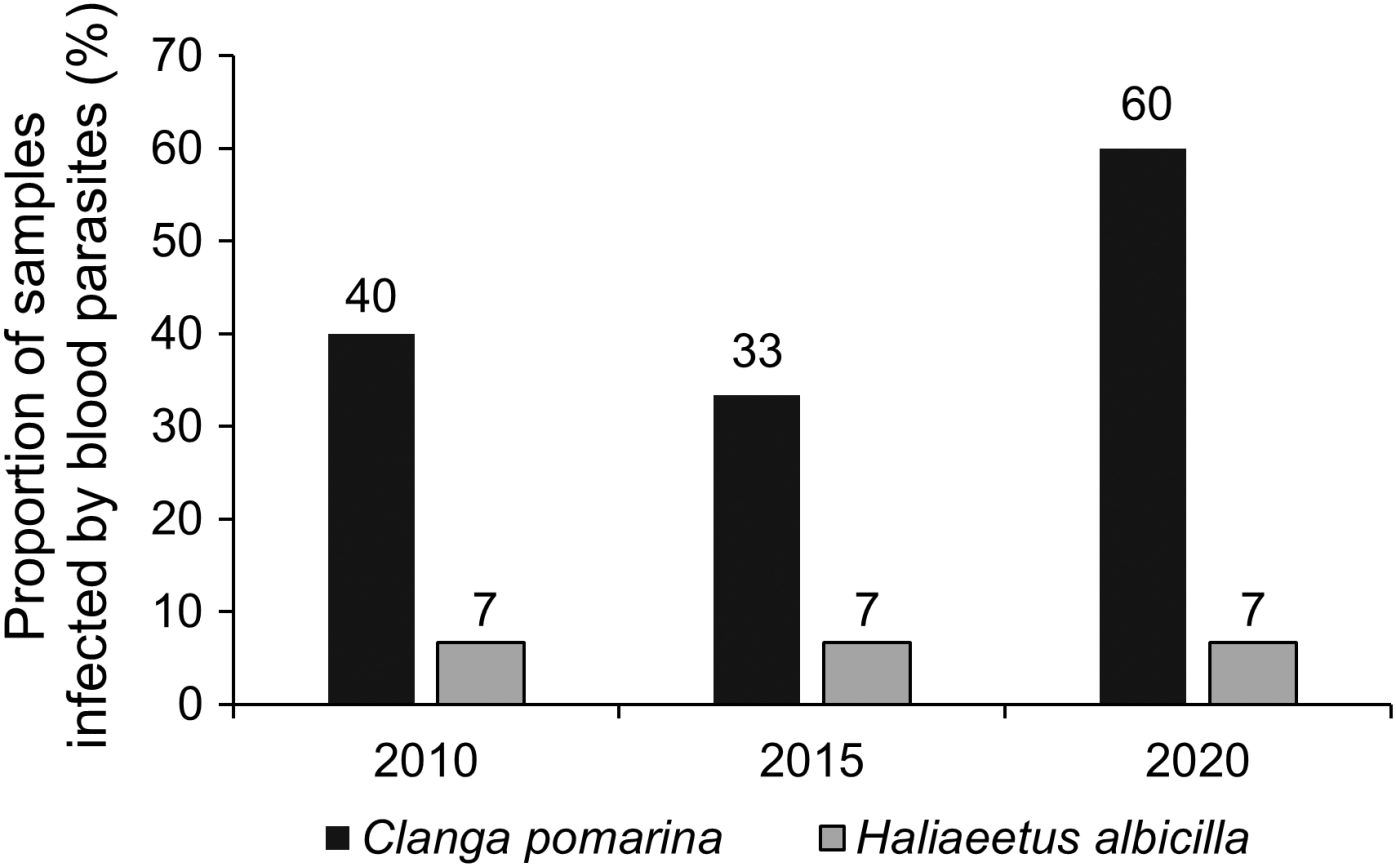
Proportion of lesser spotted and white‐tailed eagle individuals infected with blood parasites, as detected through the analyses of feathers non‐invasively sampled during the 2010, 2015 and 2020 breeding seasons. The sample size for each eagle species per breeding season was 15, resulting in a total sample size of 90 individuals.

Among resident white‐tailed eagle individuals, only 7% (*n* = 45) were infected by blood parasites, while the proportion of infected individuals among migrant lesser spotted eagles was 44% (*n* = 45) (Figure 2). We detected parasite DNA in 6 out of 15 lesser spotted eagle individuals whose feathers were collected during the summer of 2010, 5 out of 15 individuals sampled in 2015 and 9 out of 15 individuals sampled in 2020 (Figure 2). In annual samples of the white‐tailed eagle feathers, we detected single parasite infection for each year (Figure 2).

Parasite DNA could not be extracted from more than 70‐year‐old feathers of taxidermized lesser spotted eagle museum specimens.

**FIGURE 3:**
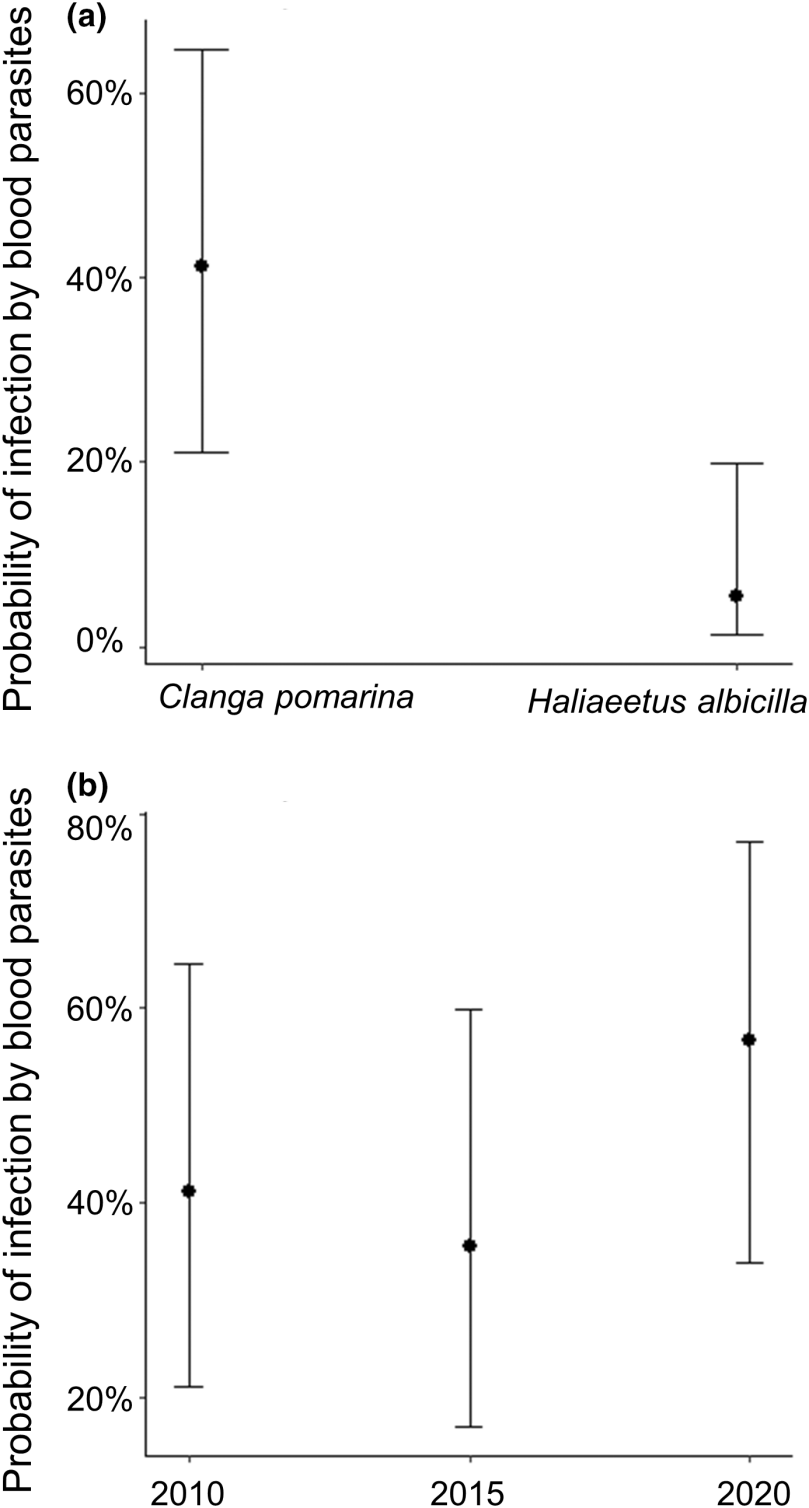
Predicted probabilities (GLM results) of blood parasite infections between two eagle species and feathers collected during the breeding seasons of 2010, 2015 and 2020. The lines (whiskers) denote 95% confidence intervals (CI).

The GLM, which examined the probability of infection by blood parasites using two explanatory variables—eagle species and the age of feathers used as a DNA source—was strongly supported by the data (i.e. ΔAICc was 13.9 lower compared to intercept‐only GLM). Accordingly, the probability of infection was significantly higher in the feathers of lesser spotted eagle individuals compared to those of white‐tailed eagles (estimate in GLM: 2.5 ± 0.7 SE, *z* value = 3.6, *p* = .0003, Figure 3a). The impact of feather age on infection probability, however, was not statistically supported. While the highest probability of infection was found in most recently (i.e. in 2020) sampled feathers, the probability of infection in feathers sampled five (−0.86 ± 0.67 SE, *z* value = −1.29, *p* = .2) or even 10 years ago (−0.63 ± 0.65 SE, *z* value = −0.96, *p* = .34) was not significantly lower (Figure 3b).

A total of nine genetic lineages, belonging to three genera, were discovered: *Haemoproteus* spp. (*n* = 4), *Plasmodium* sp. (*n* = 1) and *Leucocytozoon* spp. (*n* = 4) (Table 1). Out of these lineages, seven were determined as new, with five being detected in lesser spotted eagles and two in white‐tailed eagles. The examined lesser spotted eagles exhibited a twofold higher diversity of parasites, with six genetic lineages identified in total, compared to the three lineages identified from white‐tailed eagles. The most prevalent lineages were lCLAPOM04 and lCLAPOM06, which were found in 20% and 7% of lesser spotted eagle individuals, respectively. The remaining lineages (hLAMPUR01; hHALALB03; hCLAPOM07; hCLAPOM08; pCIAE01; lCLAPOM05; lHALALB02) were each detected only once. Additionally, among the examined lesser spotted eagle individuals, four were infected with more than one parasite (co‐infection), while no instances of co‐infection were detected in the examined white‐tailed eagle individuals.

**TABLE 1:** Results of investigated raptor birds and detected haemosporidian parasites.

Bird species	Parasite genus	Lineage name	2010	2015	2020	Total	GenBank accession no
*Clanga pomarina* (*n* = 45)	*Leucocytozoon*	lCLAPOM04	1	1	7	9	OR283229
		lCLAPOM05
			1		1	OR283230
	lCLAPOM06
	1	1	1	3	OR283231
	*Haemoproteus*	hCLAPOM07			1	1	OR283232
		hCLAPOM08	1			1	OR283233
*Plasmodium*	pCIAE01		1		1	OR283237
Co‐infection	–	3	1		4	
*Haliaeetus albicilla* (*n* = 45)	*Leucocytozoon*	lHALALB02		1		1	OR283234
	*Haemoproteus*	hLAMPUR01			1	1	OR283236
		hHALALB03
1			1	OR283235

### 3.2 Phylogenetic analysis

**FIGURE 4:**
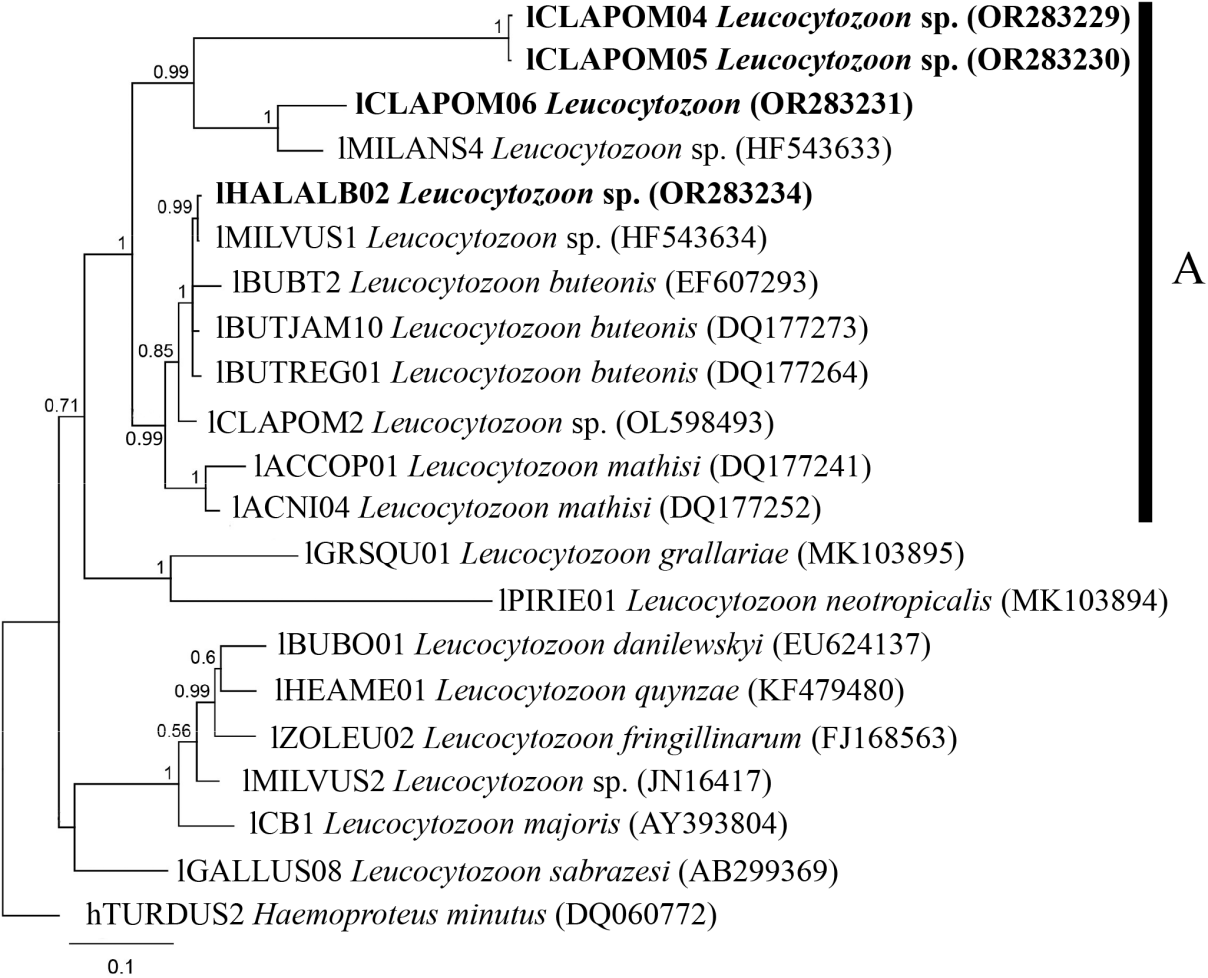
Bayesian phylogeny of the mitochondrial *cytb* lineages (478 bp) of avian *Leucocytozoon* spp. parasites. Four new lineages (indicated bold) and 16 *Leucocytozoon* spp. lineages obtained from the GenBank were used. The tree was rooted with *Haemoproteus minutus* (hTURDUS2). A‐indicates species that are known parasites of birds of prey. GenBank accession numbers are provided in parentheses.

The constructed phylogenetic tree of newly described and known *Leucocytozoon* spp. parasites illustrated that the novel genetic lineages lCLAPOM04 and lCLAPOM05 formed a well‐supported separated clade, that may represent a new morphospecies. The difference between these two lineages is 1 bp (genetic distance 0.2%) (Table 2). The genetic distance between the clustered lineages lHALALB02 and lMILVUS01 is 1 base pair (0.2%). Meanwhile, the novel lineage lCLAPOM06 developed a distinct clade by clustering with the known lineage lMILANS04 (Figure 4). The genetic distance between lCLAPOM04 and lCLAPOM06 is 36.9%. All these newly described parasite genetic lineages clustered together with other birds of prey parasites (Figure 4a).

**TABLE 2:** Results of investigated raptor birds and detected haemosporidian parasites. Genetic difference between new obtained and the closest lineages (%).

	lCLAPOM04	lCLAPOM06	lCLAPOM05	lHALALB02	lMILANS4	lMILVUS1
lCLAPOM04		–	–	–	–	–
lCLAPOM06	36.9		–	–	–	–
lCLAPOM05	0.2	36.5		–	–	–
lHALALB02	46.7	23.7	46.3		–	–
lMILANS4	36.1	10.3	35.8	21.7		–
lMILVUS1	46.0	23.3	45.7	0.2	21.3	

**FIGURE 5:**
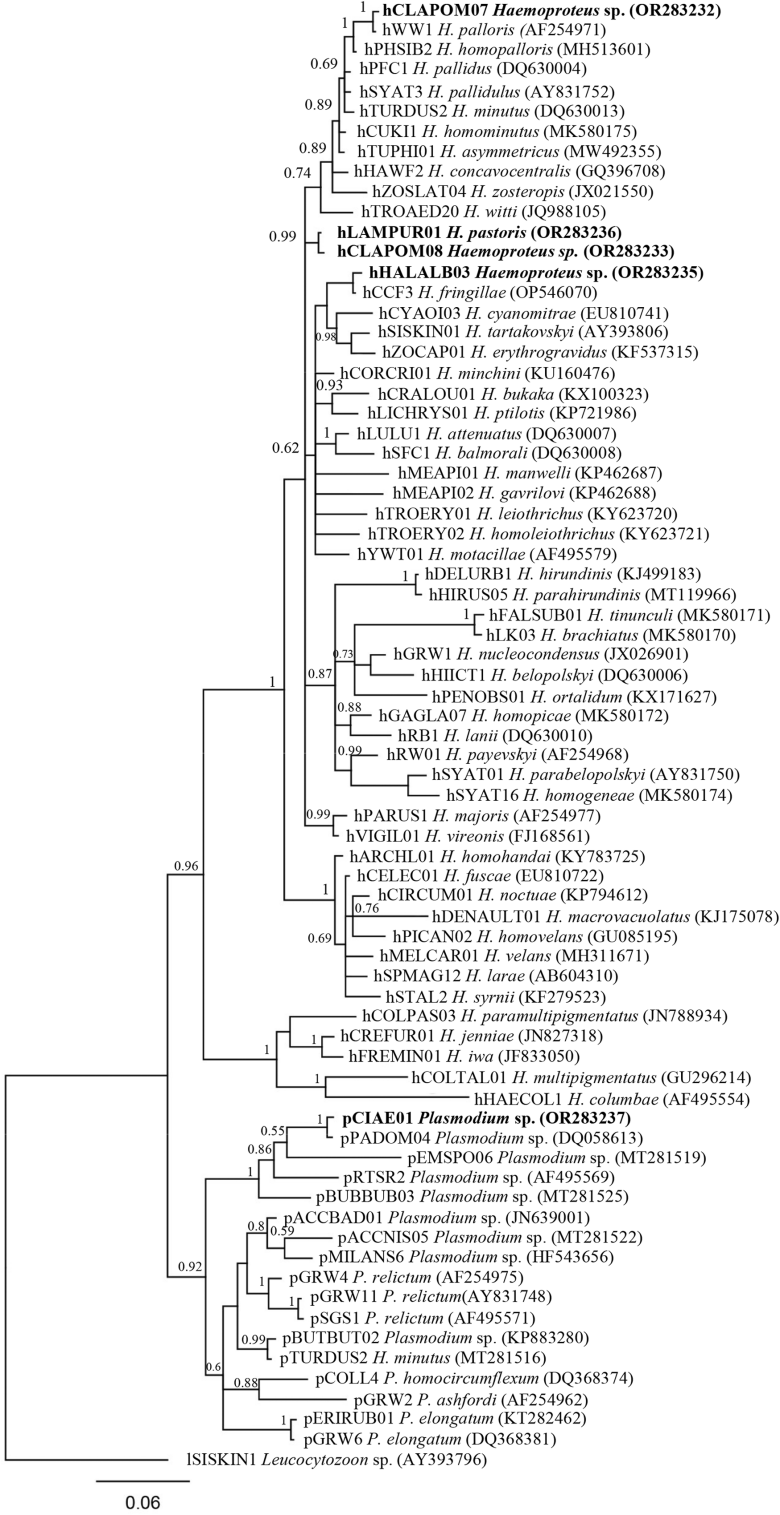
Bayesian phylogenetic tree of the mitochondrial *cytb* lineages (478 bp) of avian *Haemoproteus* spp. and *Plasmodium* spp. parasites. Sixteen *Plasmodium* spp., 51 *Haemoproteus* spp. and 5 lineages obtained from this study (indicated in bold) were used. The tree was rooted with *Leucocytozoon* sp. (lSISKIN1). GenBank accession numbers are provided in parentheses.

The constructed phylogenetic tree of newly described and known *Haemoprteus* parasites shows that hCLAPOM08 clustered with *Haemopoteus pastoris* (hLAMPUR01), differing from the latter by only one base pair (0.2%) as well as hCLAPOM07 with *H. palloris* (hWW1). Lineage hHALALB03 clustered with *H. fringillae* (hCCF3), showing a difference of 2 base pairs (0.4%). All *Haemoproteus* spp. lineages clustered with parasites primarily detected in passerine birds (Figure 5).

Only one genetic lineage belonging to genus *Plasmodium* (pCIAE01) was detected in birds of prey. This lineage clustered together with pPADOM04 with 2 bp difference (0.4%) (Figure 5).

## 4 DISCUSSION

Firstly, our study shows that dried blood extracted from the moulted feathers collected from wild bird populations is a suitable source of DNA material to study haemosporidian parasite prevalence and diversity in currently under‐researched avian groups, such as birds of prey. Secondly, we demonstrate that the DNA of haemosporidian parasites can successfully be isolated from moulted feathers of varying ages, spanning at least a decade. While DNA isolation was more successful in freshly collected feathers compared to feathers collected 5 and 10 years ago, the difference was not statistically significant. DNA isolation failed from very old feathers (≥70 years), likely due to DNA degradation and the presence of chemicals used to prevent pests in taxidermized animals. Lastly, we found that migratory lesser spotted eagles exhibited a significantly higher prevalence and diversity of haemosporidian parasites compared to resident white‐tailed eagles. A total of nine genetic lineages of haemosporidian parasites (seven of which are new to science) were detected.

Our results demonstrate marked differences in parasite prevalence and diversity between two sympatrically breeding eagle species in the temperate forests of central‐eastern Europe. Lesser spotted eagles, which migrate and overwinter in south‐eastern Africa (Meyburg, 2021), showed notably higher rates of infection compared to year‐round resident white‐tailed eagles. Although the breeding habitats of both species overlap (Treinys et al., 2011)—pairs of eagles of two species may nest in the same forests with an average distance of 1.6 km from each another (Dementavičius et al., 2019)—their exposure to similar vectors from April to September did not result in comparable infection rates. This suggests that lesser spotted eagles likely acquire infections outside of Europe, potentially during migration or within their overwintering grounds. Using a non‐invasive approach, we found that only 7% of the white‐tailed eagle and 44% of lesser spotted eagle individuals in populations were infected with haemosporidian parasites. These species have been inadequately studied due to challenges in collecting blood samples from adult birds in the wild. Notably, Krone et al. (2008) only detected haemosporidian parasites in one out of 38 white‐tailed eagle individuals (33 of which were nestlings) in Germany. Further, Harl et al. (2022) identified *Leucocytozoon* spp. parasites in both white‐tailed and lesser spotted eagles in Austria, while Nourani et al. (2020) studied these bird species in Iran and found that they were not infected with haemosporidian parasites, although the sample size was limited.

Comparing our results to global parasite prevalence rates in birds of prey, the prevalence of infection in Turkey (32%; Ciloglu et al., 2016), the United States of America (29.9%; Sehgal et al., 2006) and Germany (21.8%; Krone et al., 2008) was similar to those presented in the current study (i.e. 26% of combined sample of both eagle species). Kazakhstan, however, exhibited a much higher prevalence of infection (63.2%; Sehgal et al., 2006), potentially attributed to vector availability and/or the migratory behaviours of the bird species studied, namely *Accipiter nisus*, *Accipiter brevipes* and *Buteo buteo vulpines*. These long‐distance migrants overwinter in the areas located in southern latitudes, where vectors are abundant. Interestingly, despite this, the overall prevalence of infection in migratory lesser spotted eagles in our study remained markedly lower (44%) compared to that of Sehgal et al. (2006). However, in our most recent 2020 sampling survey, our findings (60%, *n* = 15) closely matched those presented in Sehgal et al. (2006).

Among the detected haemosporidian parasites, the lesser spotted eagle harboured all three genera, while only two genera were detected in white‐tailed eagles, namely *Leucocytozoon* and *Haemoproteus*. The lower prevalence and parasite diversity in white‐tailed eagles may be explained by the fact that this species is resident and spends its life in temperate climate areas usually within 500 km from their natal areas (Struwe‐Juhl & Grünkorn, 2007). *Leucocytozoon*, the most frequently detected genus in our study, relies on blood‐sucking black flies (Diptera: Simuliidae) for transmission (Valkiūnas, 2005). These insects are distributed worldwide, except in Antarctica, and require running water for larval development (Adler et al., 2004). The higher prevalence and diversity of *Leucocytozoon* parasites in the migratory lesser spotted eagle can be attributed to its wintering grounds in Africa, an area rich in black fly vectors (Adler et al., 2004). The presence of *Leucocytozoon* in white‐tailed eagles suggests that the transmission of these parasites also takes place in Europe and that there are European black fly species capable of transmitting *Leucocytozoon* parasites.

*Haemoproteus pastoris* (LAMPUR01), a known passerine bird parasite, was detected in the studied eagles, in addition to three new genetic lineages that are closely related to other passerine parasites. Based on the Malavi database (Bensch et al., 2009), *Haemoproteus pastoris* (LAMPUR01) and *H. fringillae* (CCF3) have previously only been found in Passeriformes birds, whereas *H. palloris* (WW1) has been detected in Anseriformes and Bucerotiformes birds. The detection of parasites in atypical hosts is not unprecedented. Moreover, sporozoites have been detected in the peripheral blood of birds, indicating that after blood‐sucking and release of sporozoites, sporozoites circulate in the blood for some time and then travel to the organs or degrade (Valkiūnas et al., 2009). Abortive development may also be the cause, a process whereby megalomeront development may commence in the organs but do not progress to the gametocyte stage (Ortiz‐Catedral et al., 2019). Given that the potential vectors of these parasites are *Culicoides* spp. (Valkiūnas, 2005), biting midges (Ceratopogoniidae) found in Lithuanian forests (Pakalniškis et al., 2006), infection could occur before feather moult.

Many birds of prey species face endangerment due to their predatory nature, persecution, sensitivity to environmental contamination and bioaccumulation of hazardous substances, as well as habitat destruction and transformation (McClure et al., 2018). Moreover, the protection of these species and their nesting grounds often involves national and international conservation efforts. Therefore, their natural scarcity in ecosystems, anthropogenic avoidance and conservation status restrict the possibilities for traditional sampling of individuals by capture in the wild. Furthermore, it has been shown that haemosporidian parasites negatively affect not only passerine birds (Donovan et al., 2008; Garvin et al., 2003) but also birds of prey (Chakarov & Blanco, 2021). Our study demonstrates that non‐invasive feather collection from nest sites is both useful and valuable, enabling larger‐scale sampling without adverse disturbance or harm. Of note, it would be valuable to develop the application of this method and to compare the repeatability of parasites detection within the different feathers of the same individuals as well as to compare detection of parasites using the moulted feathers and blood samples of the same individuals. Moreover, annual moulted feather sampling can provide insights into infection status of the same individuals in different years as well as the spatiotemporal infection dynamics at population level in relation to climate change for these long‐lived, nesting site‐tenacious birds. While PCR‐based methods are essential, combining them with microscopic examination is crucial for parasite morphological identification and lineage–species associations. This method only allows to identify the parasite's genetic lineage and whether the bird is infected with it. As the blood drop from the feather is dried, it can only be used for molecular analysis. It is not possible to perform a smear. We are therefore unable to identify the species morphologically. A comparison between the blood drawn from veins and feathers for the purpose of detecting parasites would also be interesting. However, this is research for the future. Non‐invasive methods offer a unique opportunity to investigate haemosporidian parasites of difficult‐to‐access bird groups, such as birds of prey or other protected species.

## AUTHOR CONTRIBUTIONS

**Dovilė Bukauskaitė:** Conceptualization (equal); data curation (equal); funding acquisition (lead); methodology (equal); project administration (lead); software (equal); writing – original draft (equal); writing – review and editing (equal). **Deivis Dementavičius:** Data curation (equal); writing – review and editing (equal). **Saulius Rumbutis:** Data curation (equal); writing – review and editing (equal). **Rimgaudas Treinys:** Conceptualization (equal); data curation (equal); methodology (equal); software (equal); writing – original draft (equal); writing – review and editing (equal).

## CONFLICT OF INTEREST STATEMENT

The authors declare no conflicts of interest.

## Supporting information


Table S1.

## Data Availability

The sequences were deposited in the GenBank (OR283229‐OR283237) and MalAvi databases.
